# A Bioinformatics Tool for Identifying Intratumoral Microbes from the ORIEN Dataset

**DOI:** 10.1158/2767-9764.CRC-23-0213

**Published:** 2024-02-05

**Authors:** Cankun Wang, Anjun Ma, Yingjie Li, Megan E. McNutt, Shiqi Zhang, Jiangjiang Zhu, Rebecca Hoyd, Caroline E. Wheeler, Lary A. Robinson, Carlos H.F. Chan, Yousef Zakharia, Rebecca D. Dodd, Cornelia M. Ulrich, Sheetal Hardikar, Michelle L. Churchman, Ahmad A. Tarhini, Eric A. Singer, Alexandra P. Ikeguchi, Martin D. McCarter, Nicholas Denko, Gabriel Tinoco, Marium Husain, Ning Jin, Afaf E.G. Osman, Islam Eljilany, Aik Choon Tan, Samuel S. Coleman, Louis Denko, Gregory Riedlinger, Bryan P. Schneider, Daniel Spakowicz, Qin Ma, the exORIEN Consortium

**Affiliations:** 1 Department of Biomedical Informatics, College of Medicine, The Ohio State University, Columbus, Ohio.; 2 Pelotonia Institute for Immuno-Oncology, The Ohio State University Comprehensive Cancer Center, Columbus, Ohio.; 3 Department of Human Sciences, College of Education and Human Ecology, The Ohio State University, Columbus, Ohio.; 4 Division of Medical Oncology, Department of Internal Medicine, The Ohio State University Comprehensive Cancer Center, Columbus, Ohio.; 5 Department of Thoracic Oncology, H. Lee Moffitt Cancer Center and Research Institute, Tampa, Florida.; 6 University of Iowa, Holden Comprehensive Cancer Center, Iowa City, Iowa.; 7 Division of Oncology, Hematology and Blood & Marrow Transplantation, University of Iowa, Holden Comprehensive Cancer Center, Iowa City, Iowa.; 8 Department of Internal Medicine, University of Iowa, Iowa City, Iowa.; 9 Department of Population Health Sciences, Huntsman Cancer Institute, University of Utah, Salt Lake City, Utah.; 10 Clinical & Life Sciences, M2GEN, Tampa, Florida.; 11 Departments of Cutaneous Oncology and Immunology, H. Lee Moffitt Cancer Center and Research Institute, Tampa, Florida.; 12 Department of Urologic Oncology, The Ohio State University Comprehensive Cancer Center, Columbus, Ohio.; 13 Department of Hematology/Oncology, Stephenson Cancer Center of University of Oklahoma, Oklahoma City, Oklahoma.; 14 Department of Surgery, University of Colorado School of Medicine, Aurora, Colorado.; 15 Department of Radiation Oncology, The Ohio State University Comprehensive Cancer Center, Columbus, Ohio.; 16 Department of Internal Medicine, University of Utah, Salt Lake City, Utah.; 17 Clinical Science Lab – Cutaneous Oncology, H. Lee Moffitt Cancer Center and Research Institute, Tampa, Florida.; 18 Departments of Oncological Science and Biomedical Informatics, Huntsman Cancer Institute, University of Utah, Salt Lake City, Utah.; 19 Department of Precision Medicine, Rutgers Cancer Institute of New Jersey, New Brunswick, New Jersey.; 20 Indiana University Simon Comprehensive Cancer Center, Indianapolis, Indiana.

## Abstract

Evidence supports significant interactions among microbes, immune cells, and tumor cells in at least 10%–20% of human cancers, emphasizing the importance of further investigating these complex relationships. However, the implications and significance of tumor-related microbes remain largely unknown. Studies have demonstrated the critical roles of host microbes in cancer prevention and treatment responses. Understanding interactions between host microbes and cancer can drive cancer diagnosis and microbial therapeutics (bugs as drugs). Computational identification of cancer-specific microbes and their associations is still challenging due to the high dimensionality and high sparsity of intratumoral microbiome data, which requires large datasets containing sufficient event observations to identify relationships, and the interactions within microbial communities, the heterogeneity in microbial composition, and other confounding effects that can lead to spurious associations. To solve these issues, we present a bioinformatics tool, microbial graph attention (MEGA), to identify the microbes most strongly associated with 12 cancer types. We demonstrate its utility on a dataset from a consortium of nine cancer centers in the Oncology Research Information Exchange Network. This package has three unique features: species-sample relations are represented in a heterogeneous graph and learned by a graph attention network; it incorporates metabolic and phylogenetic information to reflect intricate relationships within microbial communities; and it provides multiple functionalities for association interpretations and visualizations. We analyzed 2,704 tumor RNA sequencing samples and MEGA interpreted the tissue-resident microbial signatures of each of 12 cancer types. MEGA can effectively identify cancer-associated microbial signatures and refine their interactions with tumors.

**Significance:**

Studying the tumor microbiome in high-throughput sequencing data is challenging because of the extremely sparse data matrices, heterogeneity, and high likelihood of contamination. We present a new deep learning tool, MEGA, to refine the organisms that interact with tumors.

## Introduction

The study of microbial communities and their impact on human health has gained increasing attention over the past decade (1). The role of intratumoral microbes in the tumor microenvironment has become an increasingly important area in studying the development and progression of cancer (2). The intratumoral microbiome affects outcomes in several cancers, including *Fusobacterium nucleatum* in the development of colon cancer and *Helicobacter pylori* in stomach cancer. To explore the relationship between the microbiome and cancer, large-scale genomic datasets such as The Cancer Genome Atlas (TCGA) have been utilized. In this context, the Oncology Research Information Exchange Network (ORIEN) provides a real-world dataset consisting of clinical, genomic, and transcriptomic data collected under an Institutional Review Board (IRB)-approved common protocol known as Total Cancer Care (TCC). It represents a valuable resource for identifying intratumoral microbes from various cancer types (3). Advances in sequencing technologies have provided large-scale human tissue sequencing data, which enables the characterization of the tissue-resident metagenome. However, exploring the links between the intratumoral microbiome and cancer tissues is ongoing due to the difficulties in obtaining clinical biopsies specifically dedicated to microbial profiling.

While the interplay between cancer-specific gene–microbe interactions has garnered attention, the evolutionary underpinnings driving these interactions remain largely underexplored. The principle of evolutionary biology posits that phylogenetically related organisms frequently share analogous functional attributes, an inheritance from a common evolutionary ancestor (4, 5). Closely related species usually have similar biological functions, and they are likely to be associated with the outcome simultaneously, which suggests that closely related species often exhibit similar traits due to their shared ancestry (6–8). For instance, a study highlighted the anticancer potential inherent in specific strains of the *Streptomyces* genus in the intestinal microbiota. Intriguingly, within this genus, species composition showed nuanced variations across age brackets, alluding to the possibility that a bacterial species’ impact—be it in facilitating or suppressing cancer—could find an echo in its closely related phylogenetic kin (9). Bullman and colleagues showed the stability of the *Fusobacterium* microbiome between primary tumors and their subsequent metastases (10). Several studies emphasized the pivotal role of the *Bacteroides* genus in triggering immune-related adverse events (irAE) in immune checkpoint blockade treatments. Notably, species such as *Bacteroides vulgatus* and *Bacteroides dorei* have demonstrated predictive potential for irAEs during the immune checkpoint blockade therapy of metastatic melanoma (11–13). Moreover, the integration of phylogenetic trees in bioinformatics workflows has showcased enhanced analytic accuracy and classification robustness in analyzing host–microbiome interactions (14–16). Given these findings, there is a compelling rationale for embedding phylogenetic insights within the assessment of cancer-associated microbial communities, especially when discerning the potential significance of microorganisms within the same genus in the cancer landscape.

Here, we present Microbial Heterogeneous Graph Attention (MEGA), a deep learning–based Python package for identifying cancer-associated intratumoral microbes. The model is trained on ORIEN intratumoral microbial RNA sequencing (RNA-seq) data to identify microbial communities associated with each of the 12 human cancer types. The core framework is a heterogeneous graph transformer (HGT; ref. 17) that can learn the importance and contribution of species to cancer samples. We have shown the superior performance of HGT in characterizing cell-gene relations from single-cell multi-omics datasets (18) and identifying sample-species relations (bioRxiv 2023.04.16.537088) from The Cancer Microbiome Atlas (TCMA) data (19). To demonstrate the effectiveness and credibility of MEGA on the more complicated ORIEN data, we focus on two widely studied cancer types: colon adenocarcinoma (COAD) and thyroid carcinoma (THCA). By leveraging metabolic and phylogenetic relationships, MEGA was able to capture the association of low attention score microbes, suggesting the importance of integrating multiple types of data in identifying cancer-associated microbes. We believe that MEGA offers a comprehensive and nuanced approach to identifying cancer-associated intratumoral microbes in the ORIEN dataset, which could ultimately serve as potential targets for further study and therapy development.

## Materials and Methods

### Study Design

Established in 2014, the ORIEN is an alliance of 18 U.S. cancer centers. All ORIEN alliance members utilize a standard IRB-approved protocol: TCC. As part of the TCC, participants agree to have their clinical data followed over time, to undergo germline and tumor sequencing, and to be contacted in the future by their provider if an appropriate clinical trial or other study becomes available (20). TCC is a prospective cohort study where a subset of patients elects to be enrolled in the ORIEN Avatar program, which provides research use only-grade whole-exome tumor sequencing, RNA-seq, germline sequencing, and collection of deep longitudinal clinical data with lifetime follow-up. Nationally, over 325,000 participants have enrolled in TCC. M2GEN, the commercial and operational partner of ORIEN, harmonizes all abstracted clinical data elements and molecular sequencing files into a standardized, structured format to enable the aggregation of deidentified data for sharing across the network. Data access was approved by the IRB in an Honest Broker protocol (2015H0185) and TCC protocol (2013H0199) in coordination with M2GEN and participating ORIEN members.

### Sequencing Methods

ORIEN Avatar specimens undergo nucleic acid extraction and sequencing at HudsonAlpha or Fulgent Genetics. For frozen and optimal cutting temperature (OCT) tissue DNA extraction, Qiagen QIASymphony DNA purification is performed, generating a 213 bp average insert size. For frozen and OCT tissue RNA extraction, Qiagen RNAeasy plus mini kit is performed, generating 216 bp average insert size. For formalin-fixed paraffin-embedded (FFPE) tissue, a Covaris Ultrasonication FFPE DNA/RNA kit is utilized to extract DNA and RNA, generating a 165 bp average insert size. RNA-seq is performed using the Illumina TruSeq RNA Exome with single library hybridization, cDNA synthesis, library preparation, and sequencing (100 bp paired reads at Hudson Alpha, 150 bp paired reads at Fulgent) to a coverage of 100M total reads/50M paired reads.

### Microbe Abundance and Diversity

RNA-seq reads are used to calculate microbe abundances using the (exotic) pipeline, as described previously (3). Briefly, reads are aligned first to the human reference genome, and then unaligned reads are mapped to a database of bacteria, fungi, archaea, viruses, and eukaryotic parasites. The observed microbes then proceed through a series of filtering steps to carefully and conservatively remove contaminants before batch correction and normalization. Diversity measures were estimated by calculating the Shannon and Simpson indices, as well as Chao1, ACE, and inverse Simpson using the R package vegan.

**FIGURE 1:**
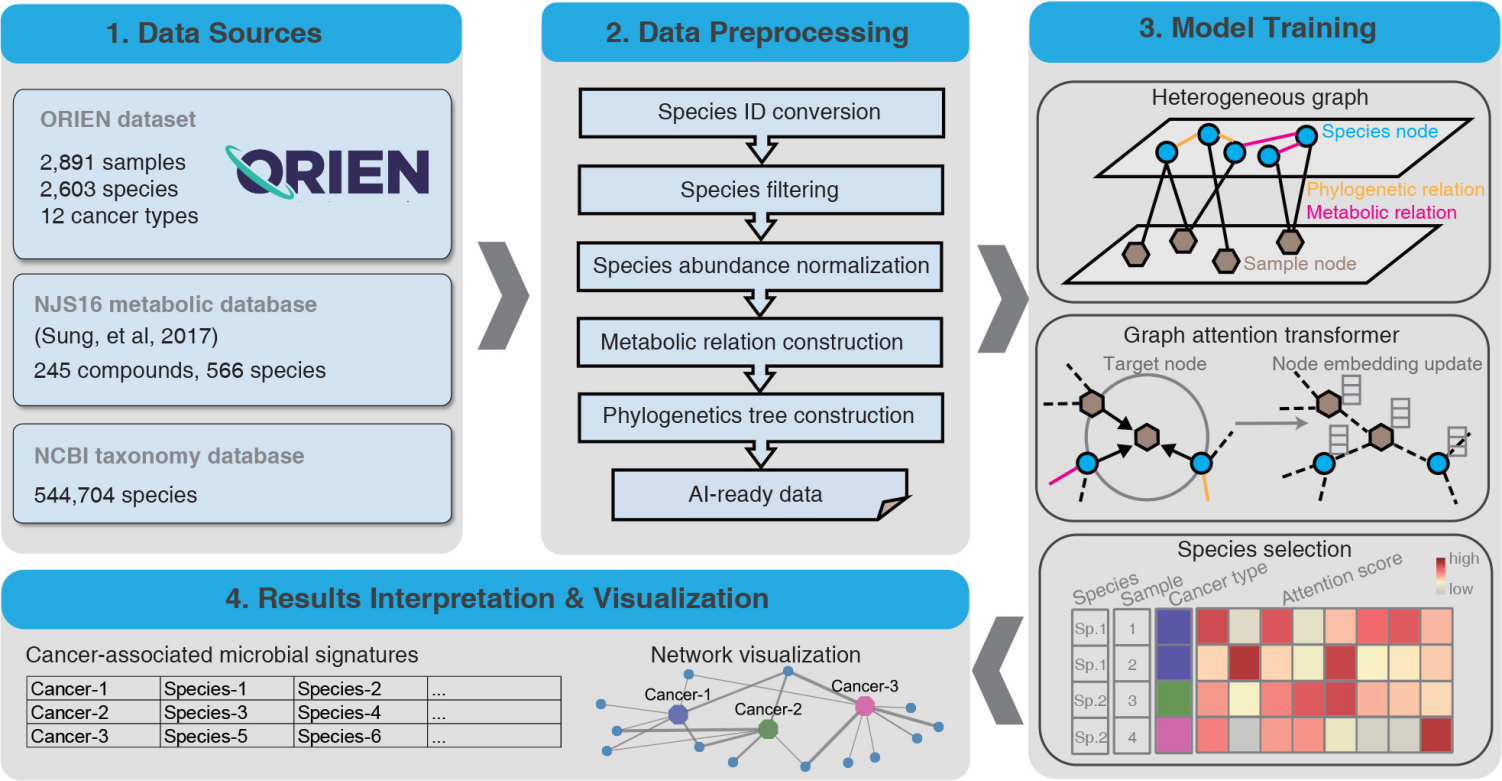
Overview of the MEGA workflow. Four main steps were included in carrying out model training and biological gene network inference. MEGA uses ORIEN datasets and two database dependencies as the data sources. Preprocessing steps are employed to generate AI-ready data for graph neural network training. After deep learning model training, the cancer-associated microbial signatures were selected on the basis of the attention scores of each species at the sample level. The final results of the identified cancer-associated microbial communities have been provided in a tabular format and are available for additional visualization.

The input dataset for MEGA includes the microbiome matrix and the sample metadata of the cancer types. The raw counts of the ORIEN microbiome matrix consist of 2,603 species in 2,891 samples. The sample metadata is a two-column matrix that describes the label of the total of 12 cancer types at each sample. The NJS16 metabolic database (21) is a literature-curated interspecies network of the human gut microbiota, composed of approximately 570 microbial species and three human cell types metabolically interacting through more than 4,400 small-molecule transport and macromolecule degradation events. We utilized the R package *taxizedb* to access the NCBI taxonomy database (22). It was integrated to prepare for the taxonomy ID to taxonomy name conversion and to extract additional phylogenetic relationships from the ORIEN data (see Fig. 1—Data Sources).

### Data Preprocessing

We initially converted the organism's name to a standard taxonomy ID using the *taxizedb* package. Species were filtered by removing those that expressed less than 0.1% of the total species. After filtering, 2,266 species were obtained. To normalize the microbiome matrix, we scaled the values in each sample of the matrix that summed to 1. This method ensures that the contribution of each feature to the total sum is proportional to its relative abundance in the sample. We used the normalized matrix as the basis for downstream analyses. Specifically, we generated the metabolic relationship network by comparing the total species list in the ORIEN matrix with the NJS16 metabolic database. In this network, an edge was placed between two species if they shared the same metabolic compound shown in the NJS16 database. We compared the total species list in the ORIEN matrix with the NCBI taxonomy database, placing an edge between two species if they share the same genus information. Finally, the processed data, including the normalized abundance matrix, metabolic relationship network, and phylogenetic relation network, served as artificial intelligence (AI)-ready data for model training (see Fig. 1).

### Model Training

#### Heterogeneous Graph and Initial Embeddings

The main MEGA model was implemented in PyTorch (23) (1) (v1.4.0) and was trained on an NVIDIA A100 graphics processing unit (GPU) for 50 epochs (∼15 minutes). We utilized our previously developed heterogeneous graph transformer model for model training (bioRxiv 2023.04.16.537088). The input graph incorporates both species and sample nodes, along with the relations among them as edges. By capturing both neighbor and global topological features among samples and species, the model was able to construct sample-sample and species-species relations simultaneously. We used two autoencoders to generate the initial embeddings for the heterogeneous graph. This allowed the representation of each node as a dense vector, which can be used as input for the deep learning model. Meanwhile, we were able to reduce the dimensionality of each species and sample, resulting in an initial embedding size of 256 dimensions for all nodes in the graph.

#### Multi-head Attention Mechanism

The complete heterogeneous graph embedding was subsequently passed to a graph attention transformer, which was trained to learn the relations between sample and species. MEGA adopts a heterogeneous multi-head attention mechanism to model the overall topological information (global relationships) and neighbor message passing (local relationships) on the heterogeneous graph. The multi-head attention mechanism is a combination of multiple independent attention processes, enabling the model to attend to different parts of the feature space differently, thereby capturing diverse aspects of the relationships in the graph (24). On the basis of grid search results, we use *h* attention heads, setting *h* = 8 as default. Each attention head calculates the attention value between each source node and target node independently. These individual attention values are then concatenated to form a comprehensive attention vector. For each attention head in each layer of the HGT, we use node type–dependent linear projection functions to map the embeddings of the source and target nodes. This results in a key vector and a query vector for each node. These vectors are then used to compute the similarity between the source and target nodes, with an edge-type–dependent matrix applied to account for different types of connections between nodes. By concatenating the multi-head attention mechanisms, we derive an attention vector for each pair of nodes. Subsequently, we collate all attention vectors from the source nodes for a specific target node. Using the softmax function normalizes these vectors so that the cumulative importance of a source node to a target node equals 1. This normalization effectively measures the contribution of a source node to a target node. This meticulous process allows the multi-head attention mechanism in our MEGA model to effectively tease apart the intricate and heterogeneous relationships within the graph, enabling the successful identification of significant microbial signatures associated with each cancer type.

#### Optimizer, Loss Function, and Hyperparameters

We used the Adam optimizer with a learning rate of 0.003 and default settings for other hyperparameters: n_hid = 128, KL_COEF = 0.00005, and THRES = 3. The Focal Loss function was used to quantify the differences between the predicted cancer type labels and true cancer type labels. The learning rate was reduced by a factor of 0.5 when the evaluation metric stopped improving for 5 epochs.

#### Microbial Signature Identification

The heterogeneous graph representation learning facilitated the embedding of samples and species simultaneously using the transformer, yielding the attention score as an important training outcome. This score represents the importance of a source node to a target node. We extracted the attention scores from source nodes spanning from species to sample. A high attention score between a given species and a sample indicates that the species was highly represented in the sample. We leveraged this information to identify microbial signatures associated with specific cancer types. We accomplished this by counting the number of samples within the cancer type for each species with high attention scores. Species with a *P* value less than 0.05 were considered to be significantly associated with the cancer type. These reliable microbial signatures were selected and served as the final output of MEGA (see Fig. 1—Model Training).

### Model Performance Evaluation

To assess the classification performance, we used accuracy, precision, recall, and the F1-score. While accuracy offers a measure based on the entire set of prediction results, precision, recall, and the F1-score are computed as averages across the 12 cancer types.



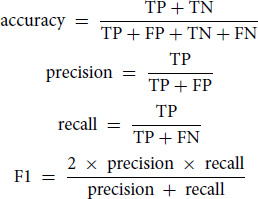



Where:

TP (true positive) = count of samples correctly classified as having the cancer
FP (false positive) = count of samples incorrectly labeled as having the cancer
TN (true negative) = count of samples correctly classified as not having the cancer
FN (false negative) = count of cancer samples incorrectly classified as not having the cancer

### Results Interpretation and Visualization

The final output of MEGA is a tab-delimited list, where each row represents each cancer type followed by identified microbial signatures. The results can be visualized in UpSet plots (25) and Cytoscape networks (26). UpSet plots are a powerful visualization technique designed to display complex set data with more than three intersecting sets. This method provides an intuitive and comprehensive means of exploring the relationships between sets and their overlaps, allowing for a more nuanced interpretation of the underlying data. Cytoscape is a widely used open-source software platform that offers a suite of tools for the visualization, analysis, and modeling of complex networks. To leverage the strengths of Cytoscape's capabilities, the RCy3 R package (refs. 3, 27) was utilized to implement the network visualization aspect of MEGA. Through the use of Rcy3’s REST application programming interface, users can seamlessly access the full feature set of Cytoscape within the R programming environment. Users can import network works directly to Cytoscape with the predefined layout and theme using MEGA output files. The network comprises cancer-species nodes, with the thickness of the edges reflecting the attention weight scores. In addition, phylogenetic or metabolic relationships between species are represented by additional edges. This approach allows for a comprehensive and nuanced exploration of the relationships between cancer and species, providing valuable insights into the underlying biological processes and pathways involved. The attention weight scores, represented by the edge thickness, highlight the key connections and interactions within the network, enabling researchers to effectively identify potential targets for further study (see Fig. 1—Results Interpretation and Visualization). Additional tutorials on generating both UpSet plots and Cytoscape networks can be found in the MEGA GitHub repository https://github.com/OSU-BMBL/MEGA.

### Implementation

MEGA was developed using Python 3.7.12 with PyTorch v1.4.0 and torch-geometric v1.4.3. The MEGA GPU mode was tested in CUDA v11.6 on a Red Hat Enterprise 7 Linux system 8.3, which featured 128-core AMD Epic central processing units (CPU), NVIDIA A100-PCIE-80GB GPUs, and 1TB RAM. Similarly, the MEGA CPU mode was tested on the Ohio Supercomputer Center Pitzer cluster, which incorporated Intel Xeon Gold 6148 CPUs and 64GB RAM. MEGA was versioned and uploaded to the Python Package Index (PyPI) using Python-Versioneer, a tool that simplifies the management of version numbers in a software project. By subjecting the software to extensive testing in both GPU and CPU modes, we ensured that MEGA functions effectively and efficiently across a range of computational architectures, ultimately providing users with a reliable and versatile tool.

### 16S Sequencing and Analysis

The bacterial 16S rRNA gene was amplified from fresh frozen tumor (*n* = 31) and adjacent normal (*n* = 31) tissues from 31 patients. Tissues were lysed on a PowerLyzer 24 at 2,000 rpm for 30 seconds, and then DNA was purified using an AllPrep mini kit (Qiagen). The bacterial rDNA was amplified using V3-V4 primers and KAPA HiFi enzyme (50°C 30 seconds, 72°C 2 × 20 cycles). Magnetic beads cleaned amplicons, and sequencing libraries were generated using a QIAseq kit (Qiagen) following the manufacturer's instructions. Libraries were sequenced on a MiSeq 2 × 300 (600 cycles) using a V3 reagent kit (Illumina). Demultiplexed fastqs were filtered for quality and length (340–440 bp). Taxonomy was assigned by processing through the precontamination filtering steps of the (exotic) pipeline v1.0.

### Plasma Metabolomics

Plasma metabolomics from 31 individuals with tumor 16S data were retrieved from the Mass Spectrometry Interactive Virtual Environment (MSV000092836). Briefly, polar metabolites were extracted in methanol, separated on a Vanquish ultra-high-pressure liquid chromatography system using an Xbridge BEH Amide (2.5 µm, 2.1 × 150 mm, Waters) column and increasing acetonitrile, as described previously (4). Ions were analyzed on a hybrid Quadrupole Orbitrap Q Exactive mass spectrometer (Thermo Fisher Scientific) in positive and negative ion modes. Compound Discoverer 3.1 1 (Thermo Fisher Scientific) was used for identification.

### Data Availability

The Ohio State University IRB approved data access through an Honest Broker protocol (2015H0185) and TCC protocol (2013H0199) in coordination with Aster Insights. The processed data generated in this study are publicly available in Gene Expression Omnibus through the BioProject PRJNA856973. The metabolomics data are available through the Mass Spectrometry Interactive Virtual Environment (MSV000092836FF).

### Code Availability

The source code and tutorial of the MEGA package have been made available under the open-source MIT license and can be freely accessed at https://github.com/OSU-BMBL/MEGA.

## Results

### MEGA Identifies Intratumoral Microbes from 12 Cancer Types in the ORIEN Dataset

**FIGURE 2:**
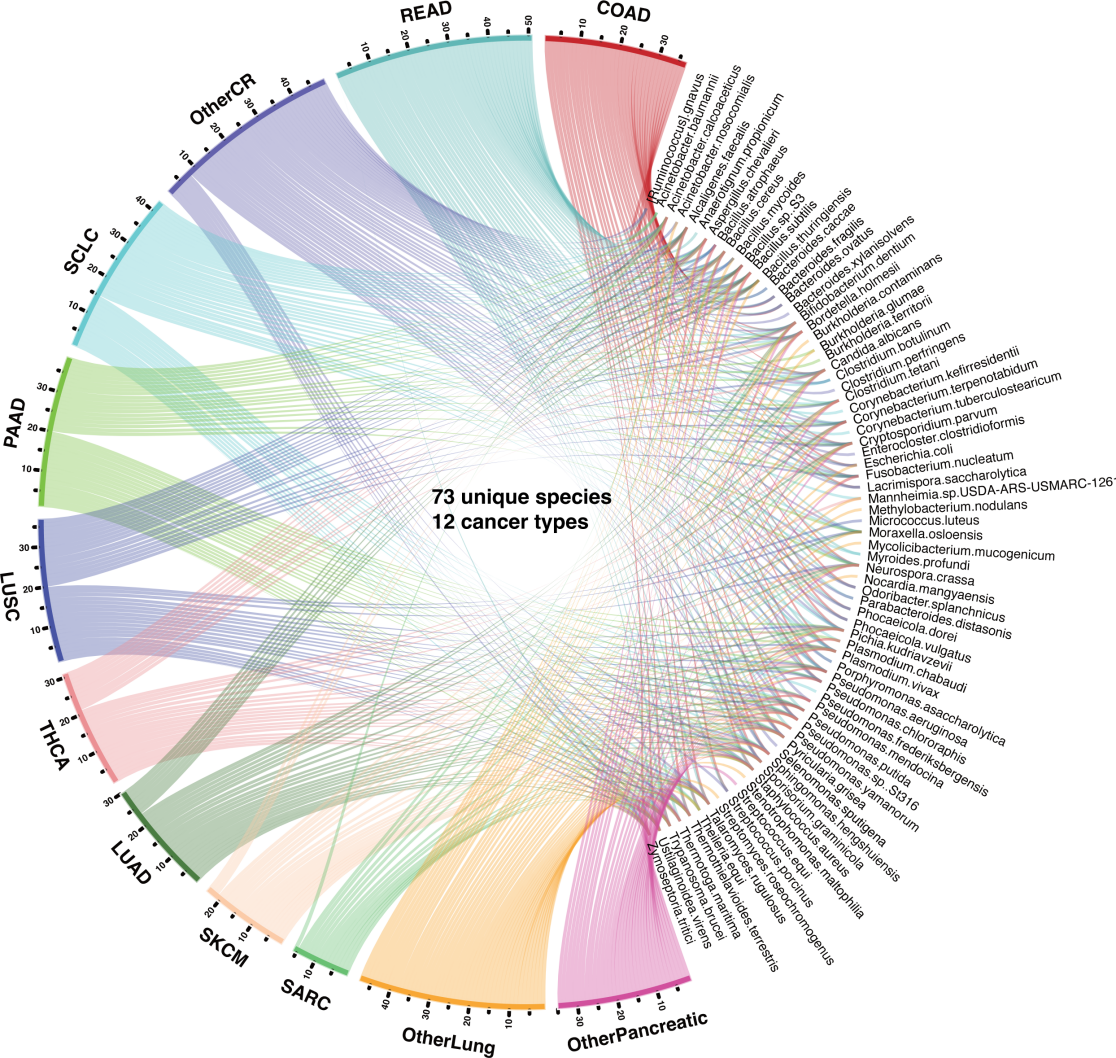
Circos plot representation of the distribution of identified species and cancer types. The segment length for each cancer type is proportional to the ratio of the total number of detected species within that cancer type, and individual ribbons are linked to their respective species. The cancer types are abbreviated as COAD (colon adenocarcinoma), LUAD (lung adenocarcinoma), LUSC (lung squamous cell carcinoma), OtherCR (other colorectal cancer types not specified), OtherLung (other lung cancer types not specified), OtherPancreatic (other pancreatic cancer types not specified), PAAD (pancreatic adenocarcinoma), READ (rectum adenocarcinoma), SARC (sarcoma), SCLC (small cell lung cancer), SKCM (skin cutaneous melanoma), and THCA (thyroid carcinoma).

Overall, MEGA is a deep learning package for identifying cancer-associated intratumoral microbes. It consists of four main steps: (i) Collect the ORIEN dataset, Human NJS16 metabolic database, and NCBI taxonomy database; (ii) Preprocess ORIEN dataset as input for the deep learning model; (iii) Train the graph attention transformer using a heterogeneous graph; and (iv) Interpret cancer-associated intratumoral microbes. Our investigation using MEGA enabled the identification of unique microbial communities comprising 73 species across 12 cancer types within the ORIEN data (Fig. 2). These findings are thoroughly tabulated in Supplementary Table S1, which provides an inclusive listing of the cancer-associated microbial signatures. Notably, certain species marked with an asterisk (*) are referenced in the literature, reaffirming their association with specific cancer types. In addition, the normalized attention weights associated with each of these identified microbial signatures are elaborated in Supplementary Table S2. Our analysis revealed that 15 species were shared across all 12 cancer types (Supplementary Table S3). Notably, eight species were uniquely shared among COAD, rectum adenocarcinoma (READ), and other colorectal cancer (OtherCR). This group of eight species represented the second-highest number of shared species across all intersections, and their shared presence is consistent with the fact that these cancers all originate in the large intestine, as in the case of colorectal cancer (see Supplementary Fig. S1). Furthermore, our study spotlighted several microbial species that exhibited associations with multiple cancer types. For example, *F. nucleatum* was identified in several cancers including COAD, Lung Adenocarcinoma (LUAD), Lung Squamous Cell Carcinoma (LUSC), READ, small cell lung cancer (SCLC), other colorectal cancer types (OTHERCR), other lung cancer types (OtherLung), and other pancreatic cancer types (OtherPancreatic). *F. nucleatum* is a gram-negative bacterium that has been widely studied for its associations with various cancers, particularly colorectal cancer (28, 29), due to its ability to promote a proinflammatory environment conducive to tumorigenesis (30). Our finding of *F. nucleatum*’s broad presence in diverse cancer types aligns with recent studies suggesting its oncogenic potential in lung (31), pancreatic (32), and colorectal cancers (33), and expands the understanding of its role in cancer beyond the traditionally associated colorectal cancer.

### MEGA Identifies Cancer-associated Microbes in COAD and THCA

To demonstrate the data analysis and interpretation capabilities of MEGA, we focused on case studies in COAD and THCA. These cancers were chosen for their contrasting levels of attention within the tumor microbiome research community. COAD has been relatively well studied in relation to its associations with tumor microbes, whereas THCA has not yet received significant attention. By using these well-known cases as a benchmark, we validated the effectiveness and credibility of MEGA. COAD is a common malignant tumor in the digestive tract (34). Increased evidence suggests intestinal microbiota was crucial in developing colorectal cancer (35). Our analysis revealed that eight microbial species were uniquely shared among the colorectal cancer types COAD, READ, and OtherCR. These species are *Bacteroides fragilis (B. fragilis)*, *Ruminococcus gnavus (R. gnavus)*, *Bacillus subtilis (B. subtilis)*, *Bacteroides ovatus (B. ovatus)*, *Lacrimispora saccharolytica (L. saccharolytica)*, *Odoribacter splanchnicus (O. splanchnicus)*, *Phocaeicola dorei (P. dorei)*, *Phocaeicola vulgatus (P. vulgatus)*, and *Streptococcus porcinus (S. porcinus)*. Notably, three of these species, *B. fragilis, R. gnavus,* and *B. ovatus,* were found to be consistent with previously validated experimental results (36–41).

**FIGURE 3:**
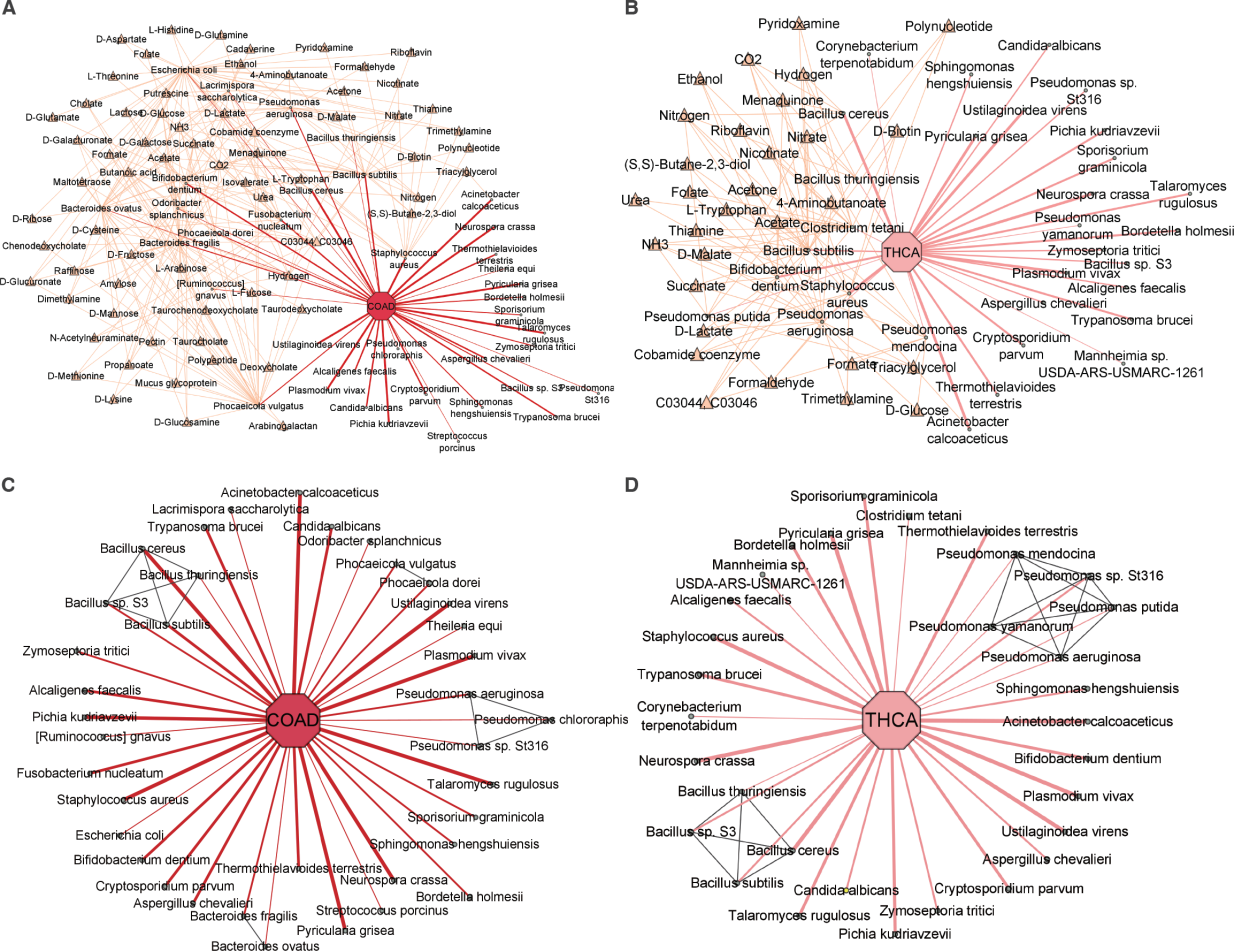
Network visualization of identified microbial communities in COAD and THCA. The cancer-type nodes were highlighted by an octagon shape, while the microbial species nodes were highlighted in a circle shape. The thickness of the edges in the network reflects the attention weight scores, indicating the strength of the relationship between the species and cancer. In addition, the metabolic compound nodes were highlighted with a yellow triangle shape, while the phylogenetic relationship edges were highlighted in gray. **A,** COAD-associated microbes highlighted with metabolic compound. **B,** THCA-associated microbes highlighted with metabolic compound. **C,** COAD-associated microbes highlighted with phylogenetic relationships. **D,** THCA-associated microbes highlighted with phylogenetic relationships.

Our model highlights the prominence of *B. fragilis* and *F. nucleatum* in COAD (Fig. 3A). These species have demonstrated oncogenic effects by modulating E-cadherin and β-catenin signaling pathways, subsequently activating proinflammatory responses (42). The influence of the *B. fragilis* toxin on colorectal cancer initiation is evident through its induction of inflammatory reactions. In addition, both *B. fragilis* and *F. nucleatum* share compounds known as short chain fatty acids (SCFAs), including butyrate, propionate, and acetate (Supplementary Table S4). While *F. nucleatum* metabolism yields high levels of SCFAs (43), these metabolites paradoxically suppress colon cancer cell proliferation. Notably, butyrate activates pyruvate kinase M2 (PKM2), a direct binding target, leading to metabolic reprogramming in colorectal cancer cells (44). This intricate interplay of microbiome and metabolites underpins the complex network within the tumor microenvironment. Dysbiosis-induced imbalances in SCFA production, influenced by diet and commensal bacteria, add further complexity (45). Other species, including *B. ovatus*, *R. gnavus*, *O. splanchnicus, L. saccharolytica*, *P. dorei*, and *P. vulgatus*, also share SCFA compounds, aligning with their roles as mediators in the communication between the intestinal microbiome and the immune system (45). The integration of metabolic relationships through MEGA reinforces its ability to decipher the intricate interplay between the microbiome and cancer.

To provide support to our findings from MEGA, we included the analysis of 16s rRNA and metabolic compounds from patients with human colorectal cancer. Interestingly, the species associated with SCFAs, as identified by our model, were corroborated in the 16s RNA dataset (*F. nucleatum*, *B. ovatus*, *R. gnavus*, *O. splanchnicus, L. saccharolytica*, *P. dorei*, and *P. vulgatus*; Supplementary Table S5). Further probing into paired metabolomics revealed the consistent presence of butanoic acid—the conjugate base of butyrate—in all analyzed samples. Butanoic acid induces apoptosis in colorectal cancer cells by connecting to the transcriptional upregulation of the Bax gene through the activation of the JNK/AP1 pathway in colonic epithelial cells (46). The presence of these microbes and SCFAs further confirms the results of our model.

*B. subtilis* and *O. splanchnicus* emerged as significant species with attention scores of 0.436 and 0.236, respectively (Fig. 3A; Supplementary Table S2). *B. subtilis* exhibited a protective effect against intestinal tumorigenesis. Conversely, the abundance of *O. splanchnicus* was diminished in patients with colorectal cancer compared with the control group (47). Further cementing its significance, our 16s rRNA data from colorectal cancer samples also confirmed the presence of *O. splanchnicus*. Our findings suggest that these bacterial species share metabolic pathways involving the compound tryptophan (48). Tryptophan, a pivotal molecule, serves as a precursor to pyridoxal 5′-phosphate (PLP), the active form of vitamin B6, participating in diverse molecular syntheses. *B. subtilis* employs the PdxST enzyme complex for PLP production (49). Notably, consistent research reveals that elevated plasma PLP corresponds to a significant reduction in colorectal cancer risk, highlighting the potential impact of these findings (50). Tryptophan and its derivates were also detected as metabolites in the colorectal cancer metabolomics dataset (Supplementary Table S6). Furthermore, the metabolic repertoire of *B. subtilis* includes riboflavin and cobalamin, each exerting distinct effects on COAD. Riboflavin displays an inverse association with colorectal cancer risk, while cobalamin is linked to an increased risk of COAD (51).

THCA has increased substantially in many countries during the past few decades (52). The species related to compound Triacylglycerol, including *Pseudomonas aeruginosa* and *Staphylococcus aureus* were found in THCA groups. Recent studies suggest that elevated triglyceride levels may be a potential biomarker for identifying individuals at a higher risk of developing thyroid cancer (ref. 53; see Fig. 3B). The full metabolic relationships for all 12 cancer types can be found in Supplementary Table S4.

By integrating phylogenetic relationships, MEGA was able to capture associations with relatively low attention scores. A previous study found that *B. ovatus* may be one of the dominant species in colon cancer (41). Although *B. ovatus* had a relatively low attention score, MEGA can identify it using the phylogenetic association with *B. fragilis*, which has a high attention score (see Fig. 3C). We found that *Pseudomonas mendocina, Pseudomonas putida,* and *Pseudomonas yamanorum* were uniquely identified in the *Pseudomonas* genus in THCA, in contrast to COAD. This aligns with the study showing the predominance of *Pseudomonas* in THCA (see Fig. 3D; ref. 54). The phylogenetic relationships for all 12 cancer types can be found in Supplementary Table S7.

## Discussion

The development of MEGA represents a significant step forward in identifying and interpreting cancer-associated intratumoral microbes. The deep learning package presented in this study utilizes RNA-seq data from the ORIEN dataset to identify microbial signatures associated with 12 different cancer types. By leveraging the power of graph attention transformers, MEGA can capture both local and global topological features of the heterogeneous graph, resulting in a more comprehensive and nuanced understanding of the underlying biological processes and pathways involved. The application of MEGA to the ORIEN dataset has provided valuable insights into the role of intratumoral microbes in cancer. The analysis revealed 73 unique species associated with the 12 cancer types studied.

Interestingly, our study identified 15 species that were shared across all 12 types of cancer examined, spanning a diverse range of both prokaryotic and eukaryotic organisms. This observation underscores the rich biodiversity within tumor microbiomes. The universal presence of these species across diverse cancer types might reflect their ubiquitous nature within the human microbiota, their adaptability to the unique conditions of the tumor microenvironment, or their potential involvement in cancer progression. For instance, *Candida albicans* and *S. aureus*, two of the shared species, have been previously associated with various forms of cancer, primarily due to their capacity to incite chronic inflammation and modulate the host cell cycle. However, it is crucial to underscore that the precise functional roles of these shared organisms across the different cancer types could be markedly different and are yet to be fully understood. Moreover, their co-occurrence across distinct cancer tissues may suggest complex interactions and adaptations between the microbiome, the tumor, and the host immune system. While our study provides a novel perspective on the common microbial signatures in cancer, further investigations are needed to elucidate the functional implications of these shared species in tumorigenesis and their potential as therapeutic targets.

While we have made considerable progress in understanding the microbiome–cancer interactions, we recognize several limitations that warrant attention. The depth of the sequencing used influences the current study's resolution, and therefore we are developing a new protocol that enhances sequencing depth for a more accurate microbial identification and abundance estimation. In addition, as we aim to integrate our results with data from sources like TCGA in the future, potential batch effects and issues related to contamination could arise. To mitigate these, stringent quality control measures are being instituted to maintain the robustness of our findings. Also, we recognize that our current methodology does not capture possible negative associations between microbial species and cancer types, an aspect we plan to explore in future investigations.

As a next step, we will further compare the cancer-associated intratumoral microbes identified from TCMA and ORIEN data using MEGA to provide a more comprehensive understanding of the role of intratumoral microbes in relation to cancer biology and host immunology. In the long run, the genotype-tissue expression (GTEx) data can be involved as control samples to identify relationships specific to tumors. In addition, applying MEGA to single-cell RNA-seq data could provide a more detailed understanding of the interactions between microbial communities and tumor cells at the cellular level. It may give us a new angle to characterize tumor heterogeneity based on intratumoral microbiome diversities. In conclusion, the development of MEGA represents an important advance in identifying cancer-associated intratumoral microbes. Our analysis of ORIEN data using MEGA revealed the presence of unique microbial signatures in specific cancer types, which may provide new targets for therapeutic intervention.

## Supplementary Material

Supplementary Figure 1 Upset plot of overall identified species in 12 cancer types.

Supplementary Table 1 Identified cancer-associated microbial signatures in all studied cancer types.

Supplementary Table 2 Identified microbial signatures with normalized attention weights.

Supplementary Table 3 Shared and unique microbial species identified across different cancer types.

Supplementary Table 4 Metabolic compounds relationships microbial signatures.

Supplementary Table 5 16s rRNA dataset from colorectal cancer samples.

Supplementary Table 6 Metabolomics data from colorectal cancer samples.

Supplementary Table 7 Phylogenetic relationships of microbial signatures.
